# Explaining cross-country variation in cigarette consumption

**DOI:** 10.1186/1617-9625-5-1

**Published:** 2009-01-09

**Authors:** Kolluru Srinivas, Bhanoji Rao

**Affiliations:** 1 Research & Analysis Department, GNN Market Research Pvt. Ltd, New Delhi, India; 2 LKY School of Public Policy, National University of Singapore, Singapore, Sri Sathya Sai University, Prasanthi Nilayam, and Administrative Staff College of India, Hyderabad, India

## Abstract

This short paper uses cross-country data on per capita cigarette consumption and selected socio-economic variables to explain inter-country differentials in consumption. It is found that the proportion of the aged in the total population and higher literacy among women have relatively greater and positive impact on cigarette consumption. Even after controlling for the effect of the two variables, a country's industrialized status has a positive impact on consumption. It would thus seem that aging and economic, and social developments are pro-cigarette consumption.

## Background

The US Surgeon General's initial report on smoking was nearly 40 years old. All these years, health warnings on cigarette packs have been in place. Despite the "warnings" and concerted efforts to dissuade potential smokers, cigarettes are here to stay. Global cigarette production and consumption have been rising steadily since cigarettes were introduced at the beginning of the 20^th ^century (see Table 1 for evidence on growth during 1960–2000). It is estimated that at present about 1.1 billion people – close to a fifth of the global population – are smokers and the number is expected to rise to more than 1.6 billion by 2025 (World Bank, 1999) [1].

**Table 1:** Global Production and Consumption of Cigarettes (in billions)

Year	Production	Consumption
1960	2150	---

1970	3112	3075

1980	4388	4328

1990	5419	5256

1995	5599	5280

1998	5581	5350

2000	5609	5489

2002	5603	5433

2003	5662	5453

2004	5530	5407

Source: US Department of Agriculture (USDA)

This paper has three main objectives: (a) to make a succinct summary of recent (1994 – 2004)^1 ^social science research on cigarette consumption, to ascertain the predominant variables affecting consumption; (b) to investigate the effects of selected socio-economic variables on per capita cigarette consumption by estimating a set of multiple regressions ; and (c) to note their implications.

### Summary of findings from social science research publications on cigarette consumption, 1994 – 2004^2^

Based on searches for refereed publications in scholarly journals via EBSCO, JSTOR, and EconLit, on the topic of cigarette consumption, we have selected 18 papers for this review and the results are summarized in Table 2. Important variables effecting cigarette consumption include prices of cigarettes, taxation, per capita income, schooling level, age, expenditure on advertisements and other promotional activities, health indicators, and anti-smoking campaigns.

**Table 2:** Main Findings of Selected Social Science Research Papers on Cigarette Consumption, 1994 – 2004

Reference	Main Findings
Keeler et al, 2004 [4]	Econometric analysis of cigarette consumption shows the negative impact of price rise. However, increased spending on advertisements offsets the effect to some extent.

Kim and Seldon, 2004 [5]	Taxation has negative impact on cigarette consumption. Anti-smoking awareness programs also have negative impact.

Ling and Glantz, 2002 [6]	Marketing plays a greater role for stronger addiction among youth. Cigarette advertisements promote regular smoking and thus increase the consumption.

Cornelia and Knight, 2002 [7]	Cigarette advertisement increased the consumption among the teenagers. Antismoking advertisements prevented cigarette advertising from promoting smoking.

Isao and Zhou, 2002 [8]	Derived hypothetically cigarette demand in Japan and studied the impact of propaganda on cigarette consumption.

Badi and Griffin, 2001 [9]	Reexamined the rational addiction models of Becker, Grossman and Murphy (BGM) for cigarette consumption. The results are supportive of the rational-addiction model.

Keeler et al, 2001 [10]	Estimates consumer response to cigarette price change (reduces the consumer response by 40–50%). Hypothesizes that a correlation between schooling and healthy behaviour occurs.

Teh-wei Hu and Yi-wen Tsai, 2000 [11]	Education and occupation are two important factors in explaining smoking in rural China. People in rural China consume fewer cigarettes than those in urban areas.

Ping Zhang et al, 2000 [12]	Tobacco price support programme (restrictions on imports and quotas) has direct (negative) effect on cigarette consumption.

Chapman et al, 1999 [13]	Australian and US restrictions on smoking at work places has the effect of reducing smoking rates and prevalence.

Depken, Craig A, 1999 [14]	Complete banning of cigarette advertising will not influence the prices of cigarettes, while limits on marketing initiatives reduce the cigarette prices.

Hsieh, Chee-Ruey, 1999 [15]	Taiwan has counterbalanced the impact of market opening on overall cigarette consumption (positive effect) by antismoking campaigns (negative effect).

Yen, Steven T, 1999 [16]	Considers two alternative models and concludes that they generate similar demand elasticities for smoking among women.

Showalter, Mark H, 1998 [17]	State excise taxes are found to be more effective in reducing cigarette consumption than federal excise taxes.

Cameron et al, 1998 [18]	This paper studies the effect of parameters like cigarette prices, income, education and health on cigarette demand. Some of the findings of earlier studies have been questioned.

George and Papapetrou, 1997 [19]	Provides an empirical analysis of cigarette consumption using the Johansen co-integration procedure.

Brown, A. Blake, 1995 [20]	The price elasticity of demand for cigarettes exports from US is estimated through the increased excise taxes, smoking restrictions, tobacco prices and quantities.

Becker et al, 1994 [21]	Empirical results are derived to indicate that smoking is addictive.

The main point about the findings in earlier research is that cigarette consumption has all the features of consumption that is addictive, and yet it is useful for policy purposes to see if any particular socio-economic variables have an important bearing on cigarette consumption in the aggregate.

### Data for our study and results of analysis

Despite persistent efforts by governmental, inter-governmental and non-governmental agents to significantly reduce cigarette consumption, the ground reality is that consumption has been on the rise. Should there be a further strengthening of the efforts aimed at reducing consumption? If so, are there any significant focus areas: specific groups of nations and age groups, for instance?^3 ^[2]Further more, given the strong finding that smoking among pregnant women could cause serious damage to the unborn (Bradford, 2003) [3], is there a likelihood of increasing consumption among women?

The aforementioned issues are addressed in this paper on the basis of cross national multiple regressions with annual per capita cigarette consumption as the dependent variable and the following as the independent variables: percent of population aged above 65 years, female literacy rate and per capita GDP in Purchasing Power Parity (PPP) Dollars. Additionally, to account for possible differential patterns among developed and developing countries, a dummy variable was included in the analysis.

Consumption data were derived from the statistics published by the United States Department of Agriculture (USDA). The data for each country on domestic cigarette consumption in million pieces was obtained from the USDA website. Consumption is estimated as production plus imports minus exports.

Cigarette consumption data are available for 90 countries. There is substantial variation in annual per capita consumption. Minimum annual consumption of cigarettes per capita was found in Rwanda (4 pieces) and the maximum in Malta (3526). The average consumption for all 90 countries put together was 806 pieces. Countries, which are close to the average, are Chile (805), Singapore (836), Malaysia (857), and Indonesia (859).

Asian Development Bank (ADB) *Key Indicators *and the World Bank *World Development Indicators *(WDI) were the sources for socio-economic indicators. Wherever there were gaps in those sources, the indicators were taken from *Human Development Indicators *issued annually by the United Nations Development Programme (UNDP). All data refer to the years 2000/2001 (averages).

Prior to proceeding with multiple regression analysis, simple correlation coefficients are presented (Tables 3 and 4) for a brief review. In the table, APCCC refers to annual per capita cigarette consumption for each country. PCGDP refers to per capita gross domestic product, POP65 to percent of total population aged 65 and over, and FEMLIT to the female literacy rate. Concepts and definitions as well as measures are fully explained in the data sources cited earlier.

**Table 3:** Correlation Matrix (Linear)

	APCCC	PCGDP	POP65	FEMLIT
PCGDP	0.566**	--		

POP65	0.843**	0.618**	--	

FEMLIT	0.602**	0.552**	0.631**	--

N	90	90	90	90

**Significant at 1 percent level; * significant at 5 percent level

**Table 4:** Correlation Matrix (Log linear)

	LOGAPCCC	LOGPCGDP	POP65	FEMLIT
LOGPCGDP	0.673**	--		

POP65	0.675**	0.626**	--	

FEMLIT	0.697**	0.709**	0.631**	--

N	90	90	90	90

**Significant at 1 percent level; * significant at 5 percent level

Per capita GDP, percentage of population above 65 years, and female literacy are all positively and significantly correlated with per capita cigarette consumption in linear and log linear specifications (Tables 3 and 4). However, an important point to note from the simple correlations is that, the explanatory variables are also correlated among themselves (linear and log linear) positively and significantly. This implies that multiple regressions are likely to be adversely affected due to multi-co-linearity, which typically leads to the explosion of standard errors and the observation of statistical insignificance of the estimated coefficients, an aspect to be re-visited in the following review of the multiple regression results.

Multiple regression equations with linear and log linear specifications and with the four explanatory variables are estimated by Ordinary Least Squares method. The results are presented in Table 5 below. In general, the linear specification is preferred since the adjusted R square is high and all the coefficients are significant.

**Table 5:** Regression Coefficients (and t-ratios) based on Data for 90 Countries Dependent Variable: Per Capita Cigarette Consumption (Avg. of 1999, 2000 and 2001)

Equation Form	PCGDP Per Cap GDP PPP$ 2001	POP65 % of Population aged 65 + 2000	FEMLIT Female Literacy Rate (%) 2000	Country Dummy (Developed = 1)	Adjusted R Square	White's Test for Hetero-scedasticity
Linear -A	-0.023* (-1.92)	121.153* (10.04)	4.982* (2.28)	650.579* (4.18)	0.76	9.28^†^

Linear -B	--	113.602* (9.81)	3.598* (1.72)	474.288* (3.71)	0.75	10.54^†^

Log linear Form	0.247 (1.68)	0.037* (3.26)	0.008* (3.34)	0.156 (1.15)	0.59	9.76^†^

* Statistically significant at 5 percent level; ^† ^Indicates absence of heteroscedasticity

Between the two linear regressions, A and B, it is clear that in A, multi-co-linearity has taken its toll on the coefficient of per capita GDP which has turned negative. The adjusted R square being about the same between A and B, one could just rely on B. Based on the linear regression B, it turns out that per capita cigarette consumption is affected positively with aging, rising female literacy and industrialization (indicated by the developed country dummy).

### Implications

The regression results have important implications for the present day developing economies. Per capita cigarette consumption will increase significantly, as those economies aim to raise their overall development status, as on-going fertility declines bring future increases in aged population proportions, and as female literacy increases.

If anything, developing countries are at a threshold of a revolutionary increase in cigarette consumption. These economies offer enormous marketing opportunities for the cigarette manufactures. Unless doubling and trebling of the efforts to control cigarette consumption are in place, there is no way to restrain consumption.

## Competing interests

The authors declare that they have no competing interests.

## Authors' contributions

All authors read and approved the final manuscript.

## Appendix

^1 ^At the time of carrying out this research in 2005, we have limited our search to the past decade and thus the period refers to 1994–2004.

^2 ^See the earlier footnote.

^3 ^See Fagan (2004), for analogous ideas.
